# Biotransformation of Deoxynivalenol by a Dual-Member Bacterial Consortium Isolated from *Tenebrio molitor* Larval Feces

**DOI:** 10.3390/toxins15080492

**Published:** 2023-08-04

**Authors:** Yang Wang, Donglei Zhao, Wei Zhang, Songxue Wang, Kai Huang, Baoyuan Guo

**Affiliations:** 1 Institute of Grain and Oil Quality and Safety, Academy of National Food and Strategic Reserves Administration, No.11 Baiwanzhuang Street, Xicheng District, Beijing 100037, China; wy@ags.ac.cn (Y.W.);; 2 School of Health Science and Engineering, University of Shanghai for Science and Technology, Shanghai 200093, China

**Keywords:** deoxynivalenol, transformation, *Devosia*, *Pseudomonas*, PQQ-dependent alcohol dehydrogenase

## Abstract

In this study, a dual-member bacterial consortium with the ability to oxidize deoxynivalenol (DON) to 3-keto-DON, designated SD, was first screened from the feces of *Tenebrio molitor* larvae. This consortium consisted of *Pseudomonas* sp. SD17-1 and *Devosia* sp. SD17-2, as determined by 16S rRNA-based phylogenetic analysis. A temperature of 30 °C, a pH of 8.0–9.0, and an initial inoculum concentration ratio of *Devosia* to *Pseudomonas* of 0.1 were optimal single-factor parameters for the DON oxidation activity of the bacterial consortium SD. Genome-based bioinformatics analysis revealed the presence of an intact PQQ biosynthesis operon (*pqqFABCDEG*) and four putative pyrroloquinoline quinone (PQQ)-dependent alcohol dehydrogenase (ADH) genes in the genomes of *Pseudomonas* strain SD17-1 and *Devosia* strain SD17-2, respectively. Biochemical analyses further confirmed the PQQ-producing phenotype of *Pseudomonas* and the DON-oxidizing enzymatic activities of two of four PQQ-dependent ADHs in *Devosia*. The addition of PQQ-containing a cell-free fermentation supernatant from *Pseudomonas* activated DON-oxidizing activity of *Devosia*. In summary, as members of the bacterial consortium SD, *Pseudomonas* and *Devosia* play indispensable and complementary roles in SD’s oxidation of DON. Specifically, *Pseudomonas* is responsible for producing the necessary PQQ cofactor, whereas *Devosia* expresses the PQQ-dependent DON dehydrogenase, together facilitating the oxidation of DON.

## 1. Introduction

Mycotoxins, a class of toxic secondary metabolites produced by some fungi, pose a significant threat to food and feed safety [1]. Deoxynivalenol (DON) is a prevalent mycotoxin found in cereals, especially maize, wheat, and barley [2], with the characteristics of high incidence and high contamination level. Due to its high thermal stability, DON is difficult to remove during food and feed processing. Consumption of DON can trigger a range of pathological reactions in humans and animals, mainly including gastrointestinal disturbances and immunotoxicity [3,4]. Therefore, there is an urgent need to develop an efficient and practical approach to eliminate DON contamination in food and feed. Numerous physical and chemical methods have been proposed to mitigate DON contamination, such as ultraviolet radiation [5], heat treatment [6], adsorption treatment [7,8], and ozone treatment [9]. However, these physicochemical approaches tend to be expensive and inefficient while also prone to secondary contamination and nutrient loss. In contrast, biological detoxification utilizes the selected microorganism and biocatalyst to mediate highly specific detoxification, thereby avoiding the drawbacks of secondary contamination and nutrient loss caused by physical and chemical treatments. As a result, the biological detoxification strategy provides a more sustainable and targeted solution to mitigate the risks associated with mycotoxin contamination in food and feed.

Research on DON biotransformation began in the 1980s. Since then, many different species of microorganisms with the ability to transform DON have been successfully isolated and identified. The majority of these microbes are pure cultures, including *Devosia* spp. [10,11], *Nocardioides* spp. [12,13], *Paradevosia shaoguanensis* DDB001 [14], Agrobacterium-Rhizobium E3-39 [15], *Pelagibacterium halotolerans* ANSP101 [16], *Sphingomonas* spp. [17,18], *Bacillus* spp. [19,20], *Marmoricola* sp. MIM116 [21], *Lactobacillus rhamnosus* SHA113 [22], *Eubacterium* sp. BBSH 797 [23], *Slackia* sp. D-G6 [24], and *Desulfitobacterium* sp. PGC-3-9 [25]. The minority are microbial consortia, such as DX100 [26], PGC3 [27], a blended culture of *Pseudomonas* sp. Y1 and *Lysobacter* sp. S1 [28], an artificial bacterial consortium comprising of *Devosia* sp. A8 and *Paracoccus yeei* A9 [29], and a mixed culture of *Pseudomonas* sp. B6-24 and *Devosia* sp. A6-243 [30]. Whether as pure or mixed cultures, these microbes transform DON primarily through oxidation or epimerization at the C3-OH group, hydroxylation at C16, de-epoxidation of the C12/C13 epoxy group, isomerization, and mineralization, thereby achieving biological detoxification [12,15,17,20,21,23]. Of all these pathways, the transformation mechanisms for the oxidation of DON’s C3-OH group and the subsequent epimerization are the most common and well-studied, especially in pure culture. Two oxidoreductases, namely, the NADP-dependent aldo-keto reductase AKR18A1 from *Sphingomonas* strain S3-4 [18] and a dehydrogenase DepA from *Devosia mutans* 17-2-E-8 [31], can catalyze the oxidative conversion of DON into 3-keto-DON. It is worth noting that DepA is dependent on the PQQ cofactor, and Ca^2+^ assists in assembling the active enzyme form. Additionally, a NADPH-dependent aldo-keto reductase DepB [32] from strain 17-2-E-8 further stereoselectively reduces 3-keto-DON to the epimer of DON, 3-epi-DON. Despite the successful isolation of several mixed cultures that transform DON, elucidating the biotransformation mechanisms of DON by mixed cultures is a challenging task due to the complexity of interactions between microorganisms and the diversity of their metabolism. As a result, the biotransformation mechanisms of DON by mixed cultures still remain unclear.

*Tenebrio molitor* larva, also known as the yellow mealworm, is a species of darkling beetle that has been studied for various applications, including as a model for toxicity and pharmacokinetic studies, as an alternative protein source, and even for the degradation of polystyrene [33,34,35]. However, there is limited information available on mycotoxin transformation by *T. molitor*. The available studies mainly focus on the high tolerance of yellow mealworms to mycotoxins, the effects of mycotoxins on the growth performance of yellow mealworms [36,37], and the analysis of mycotoxin metabolites [38]. Long-term feeding of cereal bran diets potentially contaminated with mycotoxins may promote the acclimation of mycotoxin-transforming bacterial strains in the gut of yellow mealworm larvae. Based on this assumption, Wang et al. [39] isolated a novel DON-transforming bacterial strain, *Ketogulonicigenium vulgare* D3_3, from a sample of *Tenebrio molitor* larval feces, suggesting that insect feces may be a novel resource for screening mycotoxin-transforming microorganisms. DON and its metabolites are generally not retained in insect larvae fed mycotoxin-contaminated diets but are detectable in larval feces, suggesting yellow mealworms and/or their gut microbiota may metabolize DON. However, it is unclear if *T*. *molitor* itself or its intestinal microorganisms drive this metabolism. Isolating DON-degrading microorganisms from *T. molitor* larval feces could help reveal if intestinal microorganisms contribute to DON metabolism in these insects. In conclusion, whereas *T. molitor* larvae show potential for mycotoxin transformation, current research on this subject is limited. Further studies are needed to isolate and identify specific mycotoxin-transforming bacteria from the larvae’s guts and feces, as well as to gain a deeper insight into the transformation mechanism and explore potential applications.

In this study, a dual-member bacterial consortium capable of transforming DON was isolated from the feces of *T. molitor* larvae. The effects of various factors on its transformation activity were investigated. An intact PQQ biosynthesis gene cluster and two genes encoding PQQ-dependent DON dehydrogenases were identified in *Pseudomonas* strain SD17-1 and *Devosia* strain SD17-2, respectively. The PQQ production phenotype of SD17-1 and DON-transforming enzymatic activity of the two dehydrogenases from SD17-2 were also confirmed. A cooperative interaction between *Pseudomonas* and *Devosia* in DON transformation was proposed: *Pseudomonas* sp. SD17-1 acts as a source of PQQ necessary for the dehydrogenase enzymes. Meanwhile, *Devosia* sp. SD17-2, despite its inability to synthesize PQQ, possesses DON dehydrogenase and functions as the key strain responsible for catalyzing the oxidation of DON. 

## 2. Results

### 2.1. Isolation and Identification of the DON-Transforming Bacterial Community

**Figure 1:**
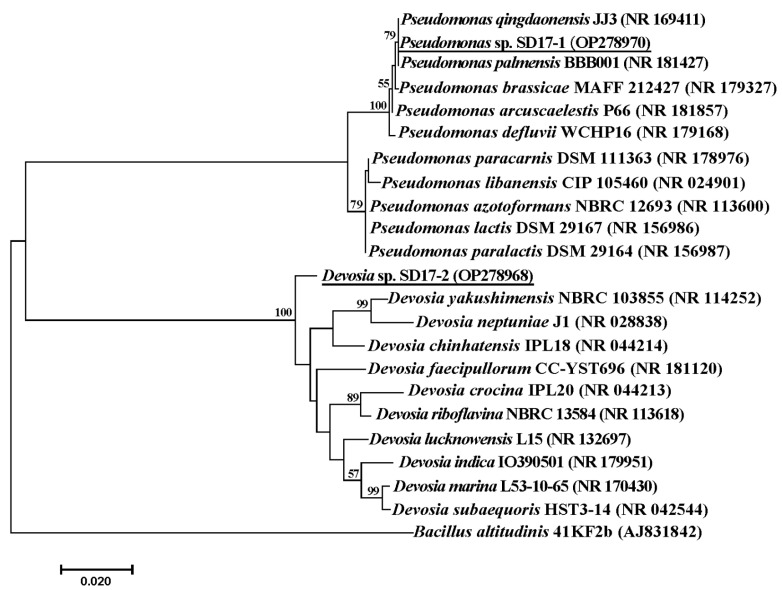
Maximum Likelihood phylogenetic tree based on 16S rRNA gene sequences indicating the taxonomic positions of strain SD17-1 and SD17-2. Sequence accession numbers are indicated in parenthesis. The two isolates obtained in this study are underlined. Numbers nearby nodes are bootstrap values (%) based on 1000 replicates. Bootstrap values greater than 50% are shown at the branching points.

Employing the enrichment culture technique, a fecal insect sample from Shandong Province, China, was enriched in MMF medium and demonstrated a remarkable ability to transform DON after four consecutive subcultures. Intriguingly, none of the individual colonies picked from TSA plate during the isolation procedure displayed DON transformation activity, necessitating the use of a mixed culture of pairwise colony combinations to obtain a microbial consortium with the desired ability. Consequently, the mixed community SD containing the two strains SD17-1 and SD17-2 was acquired. The 16S rRNA gene sequence of SD17-1 (Accession number: OP278970) displayed 98.0–100% similarity with those of the closely related type strains of the genus *Pseudomonas*, sharing the highest similarity of 100% with those of both *P. palmensis* BBB001^T^ and *P. qingdaonensis* JJ3^T^. Additionally, the 16S rRNA gene sequence of SD17-2 (Accession number: OP278968) exhibited 94.8–98.0% similarity with those of the closely related type strains of the genus *Devosia*, with the highest similarity of 98.0% with that of *D. lucknowensis* L15^T^. Phylogenetic tree analysis, shown as Figure 1, revealed that SD17-1 formed a clade with *P. palmensis* BBB001^T^ and *P. qingdaonensis* JJ3^T^, whereas SD17-2 constituted a taxon that was independent of other known species. Regarding the genomic ANIm (average nucleotide identity calculation based on MUMmer) analysis result, *Devosia* sp. SD17-1 showed ANIm values ranging from 83.52% to 84.52% when compared to the most related top 25 type strains in the genus *Devosia*, as shown in Table S1. An ANIm value below 95% indicated classification as a novel species, so SD17-1 appeared to be a novel *Devosia* species. *Pseudomonas* sp. SD17-2 showed ANIm values from 85.59% to 99.33% when compared to the top 25 *Pseudomonas* type strains. However, its ANIm values of 99.29% and 99.33% with *P. palmensis* and *P. qingdaoensis*, respectively, as shown in Table S2, exceeded the 95% threshold for species classification. Therefore, the exact species of SD17-2 could not be determined. These conclusions were consistent with the phylogenetic analysis based on 16S rRNA sequences.

### 2.2. Characterization of the Effects of Different Factors on DON Transformation Activity

**Figure 2:**
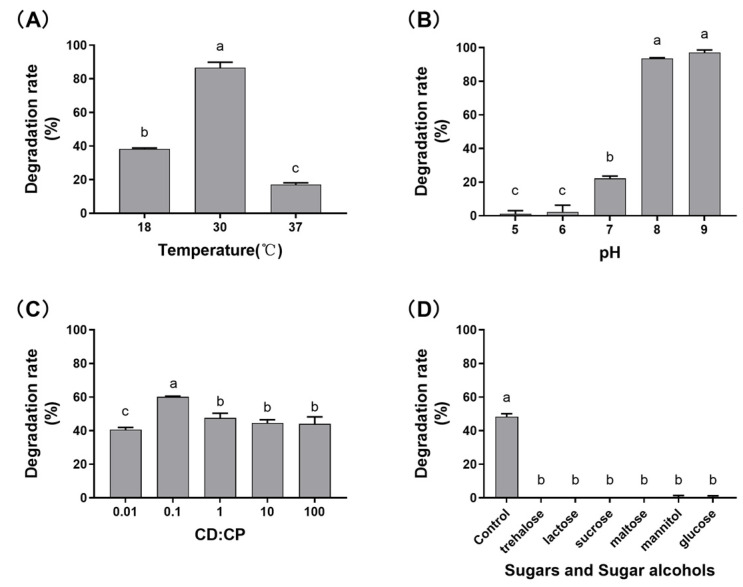
Effects of different factors on the transformation activity of DON by the bacterial consortium SD. The effects of culture temperature (**A**), pH (**B**), and inoculation ratio of *Devosia* to *Pseudomonas* (**C**), as well as sugars and sugar alcohols (**D**) on the transformation rate of DON by bacterial consortium SD. In (**C**), CD: CP indicates the initial inoculum concentration ratio of *Devosia* to *Pseudomonas*. The transformation rates’ values are indicative of the means derived from triplicate measurements, whereas the error bars serve to illustrate the standard deviations. The utilization of distinct lowercase letters signifies a significant difference (*p* < 0.05).

The impacts of temperature, initial pH, inoculation ratio of two isolates, as well as sugars and sugar alcohols on DON transformation activity of the mixed culture SD in MMF medium were investigated. The results from Figure 2A indicated that the mixed culture SD exhibited the highest transformation rate at 30 °C, with a rate of 86.6 ± 3.3%. Optimal pH values for DON transformation activity were found to be 8 and 9; however, acidic conditions (pH 5 and 6) resulted in virtually no activity (Figure 2B). Under a CFU ratios of *Devosia* to *Pseudomonas* (0.1), the mixed culture SD exhibited the highest DON transformation activity (Figure 2C). Furthermore, the addition of various sugars and sugar alcohols, such as trehalose, lactose, sucrose, maltose, mannitol, and glucose, strongly inhibited the DON transformation activity (Figure 2D).

### 2.3. Determination of DON Metabolite’ Chemical Structure

**Figure 3:**
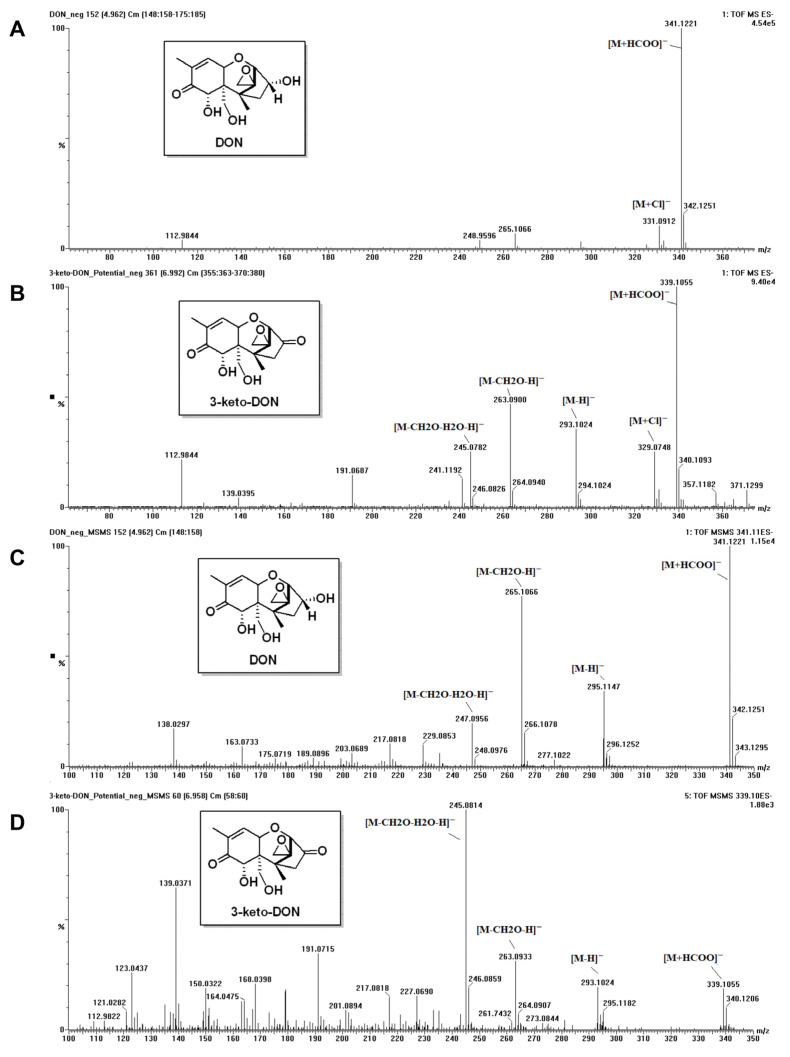
MS profiles for DON standard and its metabolite produced by the bacterial consortium SD. (**A**) Full-scan spectrum of DON; (**B**) Full-scan spectrum of DON’s metabolite; (**C**) MS/MS fragmentation profile of *m*/*z* 341.1221 of DON; (**D**) MS/MS fragmentation profile of *m*/*z* 339.1055 of the metabolite.

To identify the DON’s metabolite generated by the microbial consortium SD, we performed UPLC-MS analysis on the DON standard and the supernatant that contained the metabolite. In the negative ion mode, on full-scan spectrum, DON afforded pseudomolecular ions at *m*/*z* 341.1221, which belonged to the formate adduct ([M+HCOO]^−^, calc. 341.1242), and 331.0912 corresponded to the chlorine anion adduct ([M+Cl]^−^, calc. 331.0954) due to its distinctive isotopic pattern. For the metabolite, similar adducts were observed at *m*/*z* 339.1055 ([M+HCOO]^−^, calc. 339.1085) and 329.0785 ([M+Cl]^−^, calc. 329.0797) on its full-scan spectrum (Figure 3A,B). The exact differences in masses of formate adducts and chlorine adducts between DON and its metabolite were both two Da, which indicated that the metabolite was the oxidative product of DON with the loss of two hydrogen atoms. For further identification, an MS/MS experiment was performed to analyze the fragmentation of the formate adducts of DON and its metabolite (C, D in Figure 3). The pseudomolecular ion of DON’s formate adduct (*m*/*z* 341.1221) yielded characteristic MS2 spectrum with product ions at *m*/*z* 295.1147 ([M–H]^−^), 265.1066 ([M–CH2O–H]^−^), and 247.0956 ([M–CH2O–H2O–H]^−^). The main fragmented ions of formate adducts of DON and metabolites have been proposed and included in the Supplementary Materials (Figure S1 and Figure S2). In comparison, the fragmentation of *m*/*z* 339.1055 of the metabolite led to product ions at *m*/*z* 293.1024 ([M–H]^−^), 263.0933 ([M–CH2O–H]^−^), and 245.0814 ([M–CH2O–H2O–H]^−^). The mass differences of the characteristic product ions between DON and its metabolite were all about two Da, in accordance with the atomic mass of two hydrogens. Both MS1 and MS2 results indicated that a hydroxyl group on DON was transformed into a carboxyl group, which was in line with that in the literature [16]. Due to the stability of the possible products (15-aldehyde-DON, 3-keto-DON), the transformation mostly occurred on the 3-position of DON molecule to the 3-keto-DON. Moreover, in our MS operating condition, the metabolite of DON could readily afford a characteristic ions profile in the full-scan spectrum similar to that in its MS/MS experiment due to the in-source fragmentation. 

### 2.4. Elucidation the Underlying Mechanisms of DON Biotransformation by the Microbial Consortium SD 

#### 2.4.1. Sequencing, Assembly, and Annotation of the Genomes of Strains SD17-1 and SD17-2

To elucidate the potential mechanisms underlying the ability of the mixed culture SD, consisting of *Pseudomonas* and *Devosia*, to oxidize DON, we performed genome sequencing, assembly, and annotation of the two strains, providing a biological information basis for subsequent research. As shown as Table 1, the genome of *Pseudomonas* sp. SD17-1 had a size of 5.7 Mb with a GC content of 64.2% and contained 5170 protein-coding sequences, whereas *Devosia* sp. SD17-2 had a size of 4.2 Mb with a GC content of 62.1% and contained 4004 protein-coding sequences.

#### 2.4.2. Homology Search for PQQ Biosynthetic Gene Cluster in *Pseudomonas* sp. 17-1 and PQQ-Dependent ADH Genes in *Devosia* sp. 17-2

**Figure 4:**
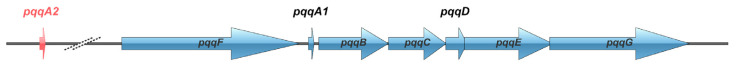
PQQ biosynthesis gene cluster structure in *Pseudomonas* sp. SD17-1.

Based on the BLASTp search results, an intact PQQ biosynthesis gene cluster was identified in the genome of the *Pseudomonoas* sp. SD17-1. The arrangement of the PQQ synthesis gene cluster in the genomic sequence of *Pseudomonas* sp. SD17-1, which contained seven open reading frames, *pqqF* (GenBank accession number of the corresponding encoding protein: WEJ21288.1), *pqqA* (WEJ24285.1 and WEJ24188.1), *pqqB* (WEJ21289.1), *pqqC* (WEJ21290.1), *pqqD* (WEJ21291.1), *pqqE* (WEJ21292.1), *and pqqG* (WEJ21293.1), was depicted in Figure 4. In particular, the gene *pqqA* encoding the PQQ precursor peptide contained two copies, *pqqA1* and *pqqA2*, and these multiple copies of the *pqqA* gene have been shown to play an important role in PQQ synthesis and to contribute differently in different growth environments [40]. Like other *Pseudomonas*, the two S9 family peptidase encoding genes *pqqF* and *pqqG* in the genome of SD17-1 were located on either side of the typical *pqqA-E* biosynthetic operon. The results suggested that strain 17-1 had the potential to produce PQQ and might play a crucial role as a PQQ provider in the DON oxidation process carried out by the microbial consortium SD. On the other hand, several essential enzymes involved in PQQ biosynthesis, namely, PqqA, PqqB, PqqC, PqqD, and PqqE, were not identified in the genome of *Devosia* sp. SD17-2, suggesting that this strain lacked the ability to produce PQQ.

To identify potential genes that encoded PQQ-dependent DON dehydrogenase, a BLASTp search was performed using DepA as the query sequence against the genomes of *Pseudomonas* sp. SD17-1 and *Devosia* sp. SD17-2. Several PQQ-dependent ADHs were found in the genomic sequence of *Pseudomonas* strain SD17-1, as presented in Table S3. However, due to their relatively low similarity and coverage with DepA, coupled with the absence of DON oxidizing capability in *Pseudomonas* cultures alone, further cloning, expression, and DON oxidation activity assays for these genes were deemed unnecessary. In contrast, the genomic sequence of *Devosia* sp. SD17-2 contained four candidate PQQ-dependent ADH genes, namely, *17dh1* (GenBank accession number of the corresponding encoding protein: WEJ32717.1), *17dh2* (WEJ32716.1), *17dh3* (WEJ31544.1), and *17dh4* (WEJ31519.1), with sizes of 1770, 1770, 1761, and 1725 bp, respectively. The corresponding proenzymes encoded by these regions comprised 589, 589, 586, and 574 amino acids, respectively. SignalP 6.0 analysis revealed that the N-terminal regions of these four enzymes contained signal peptides consisting of 1–24, 1–24, 1–25, and 1–25 amino acids, respectively. Additionally, all four enzymes belonged to the quinoprotein alcohol dehydrogenase superfamily and shared amino acid sequence homology with the DepA, with similarities of 36.9%, 36.7%, 35.9%, and 34.2%, respectively.

#### 2.4.3. Biochemical Evidence for *Pesudomonas* and *Devosia* as Producer of PQQ and DON Dehydrogenase 

**Figure 5:**
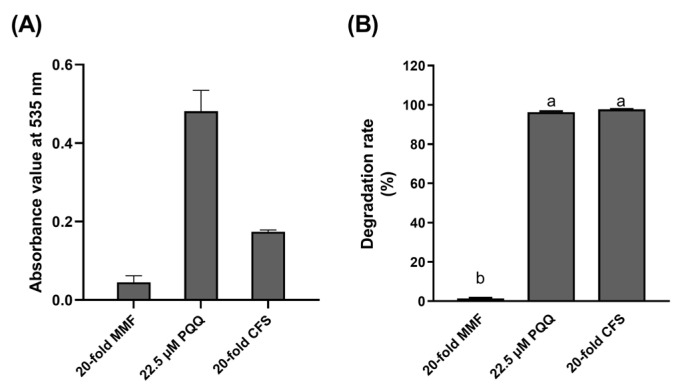
Determination of PQQ in the cell-free MMF culture supernatant of *Pseudomonas* sp. SD17-1 and its activation effect of the DON-oxidizing activity of *Devosia* sp. SD17-2. (**A**) Determination of PQQ in the cell-free MMF culture supernatant of *Pseudomonas* sp. SD17-1 using NBT method. (**B**) The activating effect of PQQ on the DON-oxidizing activity of *Devosia* sp. SD17-2. 20-fold CFS: 20-fold concentrated cell-free supernatant of *Pseudomonas*’ MMF culture. 20-fold MMF: 20-fold concentrated MMF medium. The distinct lowercase letters show a significant difference (*p* < 0.05).

The PQQ content in the 20-fold concentrated cell-free supernatant of *Pseudomonas*’ MMF culture was determined to be approximately 8.8 μmol/L using the NBT method (Figure 5A), demonstrating *Pseudomonas*’ ability to produce PQQ at the cellular level. When the supernatant was added to *Devosia* cell pellets suspension, the *Devosia* cells showed DON oxidative activity similar to *Devosia* cells treated with 22.5 μM PQQ standard; whereas *Devosia* cells supplied with 20-fold concentrated MMF medium as a control did not exhibit such activity (Figure 5B). 

**Figure 6:**
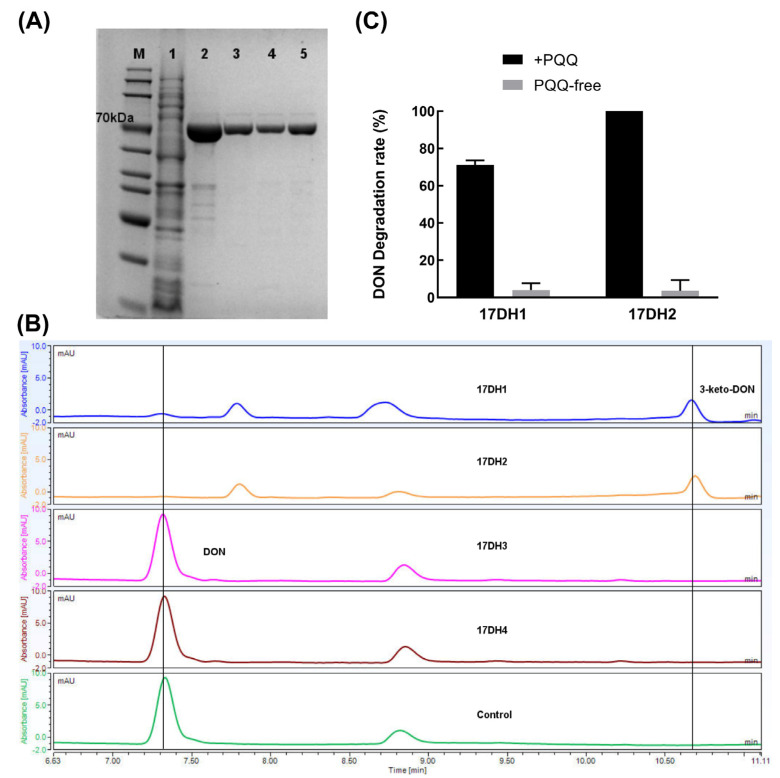
SDS-PAGE analysis, DON oxidation activity assay, and PQQ-dependence determination (**C**) of four PQQ-dependent ADHs revealed that two PQQ-dependent ADHs were responsible for DON-oxidizing activity in *Devosia* sp. SD17-2. (**A**) SDS-PAGE analysis for four PQQ-dependent ADHs. M: protein maker, 1: crude extract of *E. coli* BL21(DE3)/pET28a, 2–5: purified recombinant 17DH1, 17DH2, 17DH3, and 17DH4. (**B**) LC profiles for DON oxidation activity assay of four purified PQQ-dependent ADHs. (**C**) PQQ-dependence determination for two DON-oxidizing enzymes 17DH1 and 17DH2.

The four recombinant ADHs were heterologously expressed and then analyzed by Coomassie brilliant blue-stained SDS-PAGE. As shown in Figure 6, the approximate molecular weights of purified recombinant 17DH1, 17DH 2, 17DH 3, and 17DH 4 determined by SDS-PAGE were consistent with their respective theoretical molecular weights of 63.4, 63.4, 62.1, and 61.4 kDa. The UPLC analysis demonstrated the ability of recombinant 17DH1 and 17DH2 to transform DON, as evidenced by the complete disappearance of the DON peak and the appearance of the 3-keto-DON peak after incubation with PQQ and Ca^2+^, as well as an electron acceptor, PMS. Conversely, 17DH3 and 17DH4 did not exhibit any such activity (Figure 6). Furthermore, according to the findings of the PQQ-dependency assay, it was observed that 17DH1 and 17DH2 exhibited remarkable DON oxidation activity only in the presence of PQQ. However, in the absence of PQQ, their activity was almost disappeared, indicating that these two enzymes were strictly dependent on PQQ and functioned as DON dehydrogenases. Two PQQ-dependent DON dehydrogenases with DON oxidation activities, 17DH1 and 17DH2, exhibited amino acid sequence similarities of 36.9% and 36.7%, respectively, with DepA, whereas they shared 84.8% similarity between each other. This demonstrated the sequence diversity of PQQ-dependent ADHs with DON oxidation activity. Such sequence diversity may have accumulated through evolutionary pressures for the enzymes to adapt to the recognition and action of different substrates.

Conclusively, a cooperative mechanism can be proposed for the biotransformation of DON between *Pseudomonas* and *Devosia* bacteria based on these experimental findings. Specifically, *Pseudomonas*, which produces PQQ as a cofactor for *Devosia*’s DON dehydrogenase, acts as the PQQ supplier. *Devosia*, equipped with DON dehydrogenase, acts as the effector strain that carries out the oxidation of DON.

## 3. Discussion

Microbial pure cultures and consortia capable of transforming DON have been isolated from various environmental sources, including soil [11,13,14,15,18,20,25,26,27,28,30,41], aquatic systems [16,17], animal intestines [23,24,42], and wheat [20,21]. This suggests that microorganisms able to metabolize DON are widespread in nature. In the current study, we isolated for the first time a DON-transforming microbial consortium from yellow mealworm feces, further confirming the ubiquity of DON-transforming microorganisms. Furthermore, a novel DON-transforming bacterial strain D3_3, recently isolated from a single sample of yellow mealworm feces [39], together with the DON-transforming microbial consortia SD isolated from three additional mealworm fecal samples in the current study, suggest that yellow mealworm feces represent an unconventional yet highly efficient source for isolating mycotoxin-transforming microbes. Long-term feeding of grain feed contaminated with mycotoxins may promote the domestication of microorganisms with DON transformation capabilities in the mealworm gut, thus providing a rational explanation for the efficiency of mealworm feces as a source for screening DON transformation microbial strains.

Microbial communities with the same DON transformation mean may have different microbial compositions and vice versa. On one hand, the microbial consortia DX100 and PGC3 both have the ability to de-epoxide DON, though they differ in composition. DX100 consists mainly of *Stenotrophomonas* (80.03%), followed by *Blautia* (11.19%) and unknown microbes (3.38%), whereas PGC3 consists of 10 different genera of microbes, among which *Desulfitobacterium* may be the primary contributor to DON de-epoxidation [26,27]. On the other hand, a microbial consortium consisting of *Pseudomonas* sp. B6-24 and *Devosia* sp. A6-243 [30], which has a similar genus-level composition to the microbial community SD in this study, can transform DON into 3-epi-DON. In contrast, the microbial community SD in this study can oxidize DON to 3-keto-DON. Furthermore, we tried to analyze whether the two members of the microbial community SD with DON oxidation in this study, *Pseudomonas* sp. SD17-1 and *Devosia* sp. SD17-2, differ from *Pseudomonas* sp. B6-24 and *Devosia* sp. A6-243 at the microbial species level. Regrettably, as the 16S rRNA sequences of strains B6-24 and A6-243 have no available data in the NCBI database and related published articles, it is impossible to determine the phylogenetic relation between the *Pseudomonas* and *Devosia* bacteria identified in this study and those identified in Gao’s study [30]. Additionally, based on the ANIb analysis, *Pseudomonas* sp. SD17-1 shares 99.05% similarity with the two validly published type strains *P*. *palmensis* BBB001^T^ and *P. qingdaonensis* JJ3^T^, exceeding the 95% threshold for species classification. However, the determination of its precise classification status requires further study using the polyphasic taxonomy approach. *Devosia* sp. SD17-2 exhibits the highest similarity of 77.82% to *D. elaeis* S37^T^, suggesting it may represent a novel *Devosia* species. 

The mixed culture that consisted of *Pseudomonas* sp. SD17-1 and *Devosia* sp. SD17-2 in this study, as well as the reported microbial consortia comprising of *Devosia* sp. A8 and *Paracoccus yeei* sp. A9 [29], could both oxidize DON to the final product 3-keto-DON. However, the two microbial communities differ in their DON catalytic mechanisms. Strain A8 can oxidize DON to 3-keto-DON when cultured alone, but this capability is enhanced when it is co-cultured with electricigens such as *P. yeei* A9 or when NADPH and riboflavin are added. Researchers believed that *Devosia* sp. A8might have an NADPH-dependent aldo-keto reductase similar to AKR18A1, which catalyzes the conversion of DON to 3-keto-DON via oxidation [29]. On the other hand, *Devosia* sp. SD17-2 has two PQQ-dependent DON dehydrogenases that have the same catalytic function as DepA, but not AKR18A1-like enzymes, which are responsible for the oxidation. It is noteworthy that neither of the two microbial communities could continue to stereoselectively reduce 3-keto-DON to 3-epi-DON. This is because the absence of 3-keto-DON aldo-keto reductase similar to DepB was the reason why 3-epi-DON was not detected in the DON conversion products of the two microbial communities. 

Regarding the toxicity of 3-keto-DON, some research concludes that 3-keto-DON shows reduced toxicity compared to DON when tested on various cell lines and animal models [15,39,43]. However, in plant-based models, 3-keto-DON retains its ability to function as a potent plant toxin and has the power to inhibit plant growth [39,44], suggesting that while 3-keto-DON may be less harmful to animals, it still poses a significant threat to plants. Collectively, the microbial consortium SD harboring the capacity to enzymatically convert DON into 3-keto-DON exhibits potential for deployment in the reduction in DON in animal feed. Nevertheless, it would not be wise to pursue the development of transgenic crops via the incorporation of DON oxidizing enzyme genes from SD for resistance.

PQQ-dependent ADHs are a class of enzyme mainly present in Gram-negative bacteria, with a small number also found in eukaryotes and archaea, which catalyze the oxidation of alcohols and aldose sugars [45]. In this study, two PQQ-dependent DON dehydrogenases, 17DH1 and 17DH2, were identified from *Devosia* sp. SD17-2. The amino acid sequence similarities between 17DH1/17DH2 and DepA, a known PQQ-dependent DON dehydrogenase, were found to be 36.9% and 36.7%, respectively, indicating significant sequence diversity among PQQ-dependent ADHs with DON oxidation activities. In additional, considering the finding that the presence of sugars and sugar alcohols strongly inhibited the DON oxidation activity of the microbial community SD, and sugars and sugar alcohols may be the natural substrates of the PQQ-dependent alcohol dehydrogenase rather than DON, which is chemically a sesquiterpene derivative. Therefore, we deduce that the reason for the strong inhibitory effect of sugar alcohols on DON oxidation activity is the high concentration of sugar alcohols effectively inhibits the DON oxidation activities of the two PQQ-dependent ADHs in the effective strain SD17-2 within the microbial community through substrate inhibition effect. Although 17DH3 and 17DH4 also showed relatively high amino acid similarities of 35.9% and 34.2% with DepA, respectively, no detectable DON oxidation activity was observed for these enzymes. This suggests that factors in addition to amino acid sequence similarity may determine DON oxidation activity in PQQ-dependent ADHs. Further studies are needed to elucidate the mechanisms underlying these observations and the role of other factors in conferring DON catalytic activity. 

Although the *Pseudomonas* sp. SD17-1 and *Devosia* sp. SD17-2 strains cultured together that could synergistically oxidize DON were isolated from the yellow mealworm gut, it is still unclear whether they are indigenous or transient bacteria of the yellow mealworm gut. Additional research is necessary to determine the potential pathogenicity of *Pseudomonas* strain SD17-1 towards yellow mealworms and to assess the colonization capability of both strains in the gut of the yellow mealworm. Successful colonization would pave the way for developing a novel biological agent for detoxifying DON. Using the colonized mealworms as a biological agent could offer a simple, cost-effective, sustainable and nutritious solution for detoxifying DON-contaminated feed. These yellow mealworms colonized with DON-degrading bacteria would be likely to exhibit better DON tolerance and thus could be fed with highly DON contaminated feed, converting low-quality mycotoxin-contaminated plant protein into high-quality animal protein, thereby realizing an improvement in the utilization efficiency of mycotoxin-contaminated feed. In addition, these two PPQ-dependent alcohol dehydrogenases could potentially be modified through protein engineering to improve their activity, specificity, stability, and other properties and further developed into detoxified enzyme agents for application in feed and food industries.

## 4. Conclusions

In the present study, a microbial consortium SD with DON-transforming ability was isolated from the larval frass of yellow mealworm. The consortium consisted of two members, *Pseudomonas* sp. SD17-1 and *Devosia* sp. SD17-2, which could effectively oxidize 50 μg/mL DON into the low-toxicity metabolite 3-keto-DON within 72 h under optimal conditions. The isolation of SD from yellow mealworm frass suggested that the frass microbiome may be a source of mycotoxin-transforming microbes. The mechanism by which the bacterial consortium SD co-cultivates the oxidation of DON was elucidated based on genomic information analysis and biochemical testing. Specifically, SD17-1, a PQQ producer, provided the cofactor PQQ to SD17-2, which lacked the ability to produce PQQ but produced two PQQ-dependent DON dehydrogenases. Together, these two bacteria collaborated to achieve the oxidation of DON. Understanding the mechanism of DON oxidation by the microbial community allows for the development of strategies to optimize the biodetoxification process and reduce DON contamination. 

## 5. Materials and Methods

### 5.1. Reagent and Culture Medium 

A sterile stock solution of 10,000 μg/mL concentration was produced from DON standard procured from Pribolab of Qingdao, China. The solution was then preserved at −20 °C until its use. PQQ, phenazine methosulfate (PMS), and nitroblue tetrazolium (NBT) were purchased from Yuanye Bio-Technology (Shanghai, China). Methanol and acetonitrile of chromatography grade were purchased from Shanghai Titan Scientific Co., Ltd. (Shanghai, China). All other reagents and chemicals unless otherwise stated were from Sangon Biotech (Shanghai, China).

MMF medium was used for the enrichment and isolation of DON-transforming microorganism. It contained the following ingredients per liter of distilled water: 4.03 g of Na_2_HPO_4_·12H_2_O, 1.0 g of KH_2_PO_4_, 0.59 g of KNO_3_, 0.5 g of (NH_4_)_2_SO_4_, 0.5 g of MgSO_4_·7H_2_O, 0.019 g of CaCl_2_, 200 μL of trace element solution [46], and 100 mL of aseptic yellow mealworm feces supernatant. To prepare the aseptic insect feces supernatant, 10 g of insect feces were dissolved in 200 mL of distilled water, centrifuged, and filtered through a sterile filter to obtain a 5% (*w*/*v*) sterile solution. The other components of the MMF medium were subjected to moist heat sterilization, after which the sterile supernatant was added to the medium. 

### 5.2. Screening and Identification of the DON-Transforming Bacterial Strains 

Microbial enrichment and isolation technique was used to screen for DON-transforming microorganisms from three fecal samples of yellow mealworm collected from different regions (Beijing city, Shandong province and Fujian province) in China. For the enrichment procedure, 3 g of each fecal sample was added to 45 mL of sterile water. This mixture was vortexed for 30 min followed by 5 min of settling time. Then, 500 μL of the slurry was inoculated into 5 mL of MMF medium containing 50 μg/mL DON for cultivation. Unless otherwise stated, the cultivation conditions were at 30 °C temperature, 220 rpm rotation speed, and 3 d cultivation time. In total, 500 μL of culture was taken for sub-culturing in fresh MMF + 50 μg/mL DON medium. This process was repeated four times to obtain a stable mixed culture with DON transformation activity. For the isolation procedure, the positive mixed culture determined by UPLC analysis was diluted and spread on TSA plates, and single colonies were randomly selected for their DON transformation activities. Unfortunately, all single colonies did not show DON transformation activities. Therefore, the pure cultures were further paired and co-cultured to assess their transformation activity.

Genomic DNA of the two isolates was obtained using a bacterial genomic DNA kit (Sangon Biotech, Shanghai, China) following the manufacturer’s protocol. The 16S rRNA sequence was amplified with universal primers 27F (5′-AGAGTTTGATCCTGGCTCAG-3′) and 1492R (5′-GTTACCTTGTTACGACT-3′) on a Biometra GmbH Thermocycler (Göttingen, Germany). The PCR product was sequenced by Bomaide Gene Technology Co., Ltd. (Beijing, China). The resulting 16S rRNA sequence was analyzed by BLAST search in the NCBI database (http://www.ncbi.nlm.nih.gov/, accessed on 21 April 2023), and a phylogenetic tree was constructed by MEGA 7.0 (MEGA, Auckland, New Zealand) using the Maximum Likelihood method with 1000 bootstrap replicates. 

To determine the taxonomic status of the two isolates at the species level, we conducted a genome-based taxonomic analysis using average nucleotide identity (ANI) calculations. The genomes of isolates SD17-1 and SD17-2 obtained from Section 5.5.1 of the materials and methods were individually uploaded to the JSpeciesWS web service at https://jspecies.ribohost.com/jspeciesws/#home, accessed on 30 July 2023. Through tetra correlation search (TCS), the top 25 most closely related congeneric genome sequences were retrieved from the genomic database for each query genome. Average nucleotide identity calculations based on MUMmer (ANIm) were then performed between the query genomes and the retrieved genomes. The resulting ANIm values were used to determine the taxonomic status of isolates SD17-1 and SD17-2 according to established phylogenetic thresholds on the JSpeciesWS website.

### 5.3. Analysis of DON and Its Metabolite

#### 5.3.1. Ultra-Performance Liquid Chromatography (UPLC) Analysis for DON

Residual DON analysis was performed by a Thermo UltiMate3000 UPLC-DAD system (Waltham, MA, USA). Briefly, 2 μL of sample was injected onto an ACQUITY UPLC^®^ BEH C18 column (2.1 × 100 mm, 1.7 μm; Waters), and DON was detected using the following parameters: 220 nm wavelength, 40 °C temperature, 0.2 min/L flow rate, and a elution gradient program: 0–6 min, gradient 5–25% acetonitrile in water (*v*/*v*); 6–12 min, isocratic 25% acetonitrile; 12–13 min, gradient 25–5% acetonitrile; 13–18 min, and isocratic 5% acetonitrile. All analyses were performed in triplicate. The DON transformation rate was calculated utilizing the subsequent equation:

Dr = (1 − Dt/Dc) × 100%

In this equation, Dr represents the DON conversion rate, and Dt and Dc represent the integrated area of the absorption peak of residual DON at a wavelength of 220 nm for the treated group and the control group, respectively.

#### 5.3.2. MS (Mass Spectrometry) Analysis of DON Metabolite

The analysis of DON and its metabolite generated by the microbial community SD was conducted utilizing an Acquity UPLC combined with an Xevo G2-S quadrupole time-of-flight (Q-TOF) mass spectrometer that was furnished with an electrospray ionization source (Waters, Medford, MA, USA). The liquid chromatographic separation was executed on an ACQUITY UPLC BEH C18 column (100 mm × 2.1 mm, 1.7 μm) at 40 °C. The injection volume was 1 μL. The mobile phase consisted of two solutions: A, an aqueous solution containing 5 mmol/L ammonium formate and 0.1% (*v*/*v*) formic acid, and B, methanol; the flow rate was 0.2 mL/min, and the gradient elution program was 10% B, 0–1 min; 10–90% B, 1–19 min; 90% B, 19–24 min; 90–10% B 24–25 min; and 10% B, 25–30 min. The Q-TOF-MS was performed in the negative mode under following the optimum parameters: capillary voltage, 2.0 kV; desolvation gas (N_2_), 800 L/h; desolvation temperature, 450 °C; source temperature, 120 °C; and cone gas (N_2_), 50 L/h. Mass spectra were acquired at full-scan mode with a *m*/*z* range of 100–800. Argon was used as the collision gas and the collision energy range was 10–40 eV. Data were analyzed using MassLynx version 4.1 (Waters, Milford, MA, USA).

### 5.4. Effects of Various Factors on DON Transformation Rate 

In a transformation system, 2 mL MMF medium containing 50 μg/mL DON, the effects of different temperatures (18 °C, 30 °C, and 37 °C), various initial pH values (5.0, 6.0, 7.0, 8.0, and 9.0), the addition of different types of sugars and sugar alcohols (2% *w*/*v* of D-trehalose, lactose, sucrose, maltose, D-mannitol, and glucose), and varying CFU ratios of the dual-member microbial consortium inoculum (*Devosia*: *Pseudomonas* = 1:100, 1:10, 1:1, 10:1, and 100:1) on the DON transformation activity of the microbial consortium were investigated. Residual DON and DON transformation rates were measured and calculated, respectively, according to the Materials and Methods described in Section 5.3.1. The experiments were conducted in triplicate, and the presented transformation rate values represented the mean of three replicates, with the error bars indicating the standard deviations. To compare between multiple groups, an analysis of variance (ANOVA) was carried out, followed by pairwise comparisons using Tukey’s test (*p* < 0.05).

### 5.5. Elucidation of the Mechanism of DON Degradation by Microbial Community SD

#### 5.5.1. Genomic Sequencing and Analysis

In Biomaker Technology Inc., located in Beijing, China, the genomes of *Devosia* strain SD 17-2 and *Pseudomonas* strain SD 17-1 underwent sequencing through a combination of the Nanopore PromethION 48 system (Oxford, Oxon, UK) and the Illumina NovaSeq 6000 platform (San Diego, CA, USA). Further technical information on the sequencing, assembly, and annotation processes can be found in a previous report by Wang and Wang [47]. Subsequently, BLASTp was used with an E value of 10^−5^ by TBtools version 1.098769 [48] to search for candidate DON-oxidizing encoding genes in the genomic sequences of *Devosia* strain SD17-2 using DepA (known PQQ-dependent DON dehydrogenase, GenBank accession: KFL25551.1) from *D. mutans* 17-2-E-8 as a query sequence. Similarly, the known PQQ biosynthesis enzymes PqqA, PqqB, PqqC, PqqD, and PqqE from *Pseudomonas* were used as queries to search for an intact PQQ biosynthesis gene cluster in the genomes of *Pseudomonas* strain SD17-1, with the same method and parameter set applied as for the DON-oxidizing enzyme search. Diagram of PQQ biosynthesis gene cluster arrangement was drawn by IBS 2.0 [49].

#### 5.5.2. Identification of Two PQQ-Dependent DON Dehydrogenases from *Devosia* sp. SD17-2

Using Q5 High-Fidelity DNA Polymerase (NEB, America), we amplified four possible genes for DON oxidation and linearized pET28a from the genomic DNA of strain SD17-2 and expression vector pET28a, respectively. The PCR conditions and primers used are listed in Table S4. The resulting amplicons for the candidate gene and the linearized pET28a were individually ligated using the T5 exonuclease-dependent DNA assembly (TEDA) method [50] and then transformed into *E. coli* BL21(DE3) for expression as N-terminal His6-fusion protein. The expression and purification of recombinant proteins was performed using a previously reported method [51]. Subsequently, the purified proteins were concentrated, analyzed by Coomassie blue-stained SDS-PAGE, and stored at −80 °C until required.

To test the DON transformation activity of the four purified recombinant enzymes, reaction mixtures contained 50 μg/mL of DON, 40 μM of PMS, 1 mM of CaCl_2_, 100 μM of PQQ, and 50 nM of purified recombinant enzyme in 25 mM Tris-HCl buffer (pH 8.0). These samples, including a control without enzyme, were incubated for 2 h at 30 °C and then subjected to UPLC analysis for DON.

#### 5.5.3. NBT Assay for PQQ Produced by *Pseudomonas* sp. SD17-1

The cell-free supernatant of a 10 mL culture of *Pseudomonas* sp. strain SD7-1 grown in MMF medium for 3 d was prepared by centrifugation and filtration. The supernatant was freeze-dried to a powder, reconstituted in 500 μL of sterile water, and centrifuged. The resulting supernatant (20-fold CFS) was analyzed for PQQ content using NBT assay [30]. Briefly, 45 μL of the concentrated sample was added to a reaction mixture consisting of 25 μL of 3.6 mM NBT solution, 40 μL of 20 mM phosphate-buffered saline (pH 7.0) and, 90 μL of 20 mM glycine–potassium hydroxide buffer (pH 10.0) and incubated in the dark at 30 °C for 1 h. The absorbance at 535 nm was then measured. Positive and negative controls were 22.5 μM PQQ standard and 20-fold concentrated MMF medium, respectively.

#### 5.5.4. Activation of DON Oxidation Activity of *Devosia* sp. SD17-2 by PQQ Supplement

An aliquot of 100 μL of the aforementioned 20-fold CFS was added to a 400 μL reaction mixture containing a working concentration of 50 μg/mL DON, 1 mM CaCl_2_, 6.1 × 10^7^ CFU/mL of *Devosia* sp. SD17-2, and 25 mM Tris-HCl buffer (pH 8.0). Reaction mixture supplemented with 20-fold MMF served as negative control, whereas that supplemented with 22.5 μM PQQ standard served as positive control. These mixtures were incubated at 30 °C for 48 h and then subjected to UPLC assay for DON.

## Figures and Tables

**Table 1:** Genome summary table of *Pseudomonas* sp. SD17-1 and *Devosia* sp. SD17-2.

	*Pseudomonas* sp. SD17-1	*Devosia* sp. SD17-2
Genome coverage	180.0×	261.0×
Contig Length (bp)	5,727,087	4,231,113
Type	chromosome	chromosome
Topology	circular	circular
Genes	5249	4152
Protein-coding	5170	4004
GC content (%)	64.25	62.11
rRNA number	19	12
tRNA number	73	58
Assembly accession number	GCA_029201585.1	GCF_029201565.1

## Data Availability

The complete genomic sequences of *Pseudomonas* sp. SD17-1 and *Devosia* sp. SD17-2 have been deposited in NCBI/GenBank. Each strain has its own unique BioProject number, GenBank accession number, and BioSample number: PRJNA875557, GCA_029201585.1, and SAMN30622627 for strain SD17-1 and PRJNA874206, GCA_029201565.1, and SAMN30543117 for strain SD17-2, respectively. The 16S rRNA genes of strain SD17-1 and SD17-2 have been deposited under GenBank accession numbers OP278970 and OP278968, respectively. Additionally, data supporting the findings of this work are available within the paper and its Supplementary Information.

## Supplementary Materials

The following supporting information can be downloaded at: https://www.mdpi.com/article/10.3390/toxins15080492/s1, Table S1: The genomic ANIm analysis of *Devosia* sp. SD17-2 against the top 25 most closely related type strains in the genus of *Devosia*. Table S2: The genomic ANIm analysis of *Pseudomonas* sp. SD17-1 against the top 25 most closely related type strains in the genus of *Pseudomonas*. Table S3: Several putative PQQ-dependent dehydrogenases in *Pseudomonas* sp. SD17-1. Table S4: Primer sequence and PCR reaction program. Figure S1: The proposed product ions in the fragmentation of *m*/*z* 341.1221 ([M+HCOO]^−^) for DON. Figure S2: The proposed product ions in the fragmentation of *m*/*z* 339.1055 ([M+HCOO]^−^) for DON metabolite.
