# Supplementary material for: Biotransformation of Deoxynivalenol by a Dual-Member Bacterial Consortium Isolated from Tenebrio molitor Larval Feces
Source: Toxins (Basel). 2023 Aug 4;15(8):492. doi: 10.3390/toxins15080492 (PMC10467086; doi:10.3390/toxins15080492)
Supplement: Supplementary file 1 [file toxins-15-00492-s001.zip › toxins-2500021-supplementary.pdf]

Supplementary Materials: Biotransformation of Deoxynivalenol by a Dual-Member Bacterial Consortium Isolated from Tenebrio molitor Larval Feces

Yang Wang, Donglei Zhao, Wei Zhang, Songxue Wang, Kai Huang, Baoyuan Guo

Table S1. The genomic ANIm analysis of *Devosia* sp. SD17-2 against the top 25 most closely related type strain in the genus of *Devosia*

Genome	ANIm [%]	Aligned [%]	Aligned [bp]	Total [bp]
Devosia equisanguinis CIP 111628 [T]	84.52	23.89	1010636	4231113
Devosia sediminis MSA67 [T]	84.48	22.39	947407	4231113
Devosia riboflavina IFO13584 [T]	84.40	25.15	1064115	4231113
Devosia lucknowensis L15 [T]	84.38	18.17	768916	4231113
Devosia salina SCS-3 [T]	84.38	21.97	929724	4231113
Devosia faecipullorum CC-YST696 [T]	84.19	18.84	797347	4231113
Devosia soli GH2-10 [T]	84.18	20.39	862514	4231113
Devosia chinhatensis IPL18 [T]	84.16	18.54	784561	4231113
Devosia ureilytica XJ19-45 [T]	84.02	18.66	789556	4231113
Devosia rhizoryzae LEGU1 [T]	84.02	16.94	716714	4231113
Devosia oryzisoli PTR5 [T]	83.91	14.70	621975	4231113
Devosia ginsengisoli Gsoil 520 [T]	83.91	19.85	839792	4231113
Devosia aurantiaca H239 [T]	83.85	17.36	734430	4231113
Devosia limi DSM 17137 [T]	83.85	15.75	666210	4231113
Devosia marina L53-10-65 [T]	83.84	17.30	732099	4231113
Devosia limi DSM 17137 [T]	83.82	15.47	654724	4231113
Devosia epidermidihirudinis E84 [T]	83.78	13.90	588292	4231113
Devosia subaequoris HST3-14 [T]	83.72	16.29	689360	4231113
Devosia subaequoris DSM 23447 [T]	83.71	16.37	692686	4231113
Devosia oryziradicis G19 [T]	83.71	14.87	629234	4231113
Devosia naphthalenivorans CM5-1 [T]	83.65	13.83	584972	4231113
Devosia yakushimensis NBRC 103855 [T]	83.61	17.27	730724	4231113
Devosia psychrophila Cr7-05 [T]	83.56	15.02	635504	4231113
Devosia beringensis S02 [T]	83.53	17.13	724836	4231113
Devosia psychrophila CGMCC 1.10210 [T]	83.52	14.94	632057	4231113

Note: The "T" in square brackets represents the type strain.

Table S2. The genomic ANIm analysis of *Pseudomonas* sp. SD17-1 against the top 25 most closely related type strain in the genus of *Pseudomonas*

Genome	ANIm [%]	Aligned [%]	Aligned [bp]	Total [bp]
Pseudomonas qingdaonensis JJ3 [T]	99.33	93.00	5326121	5727087
Pseudomonas palmensis BBB001 [T]	99.29	94.04	5385877	5727087
Pseudomonas aegrilactucae MAFF 301350 [T]	89.43	73.08	4185072	5727087
Pseudomonas brassicae MAFF 212427 [T]	89.38	68.28	3910236	5727087
Pseudomonas faucium BML-PP048 [T]	86.11	47.54	2722377	5727087
Pseudomonas muyukensis COW39 [T]	86.11	47.24	2705675	5727087
Pseudomonas peradeniyensis BW13M1 [T]	86.09	47.93	2745252	5727087
Pseudomonas parasichuanensis BML-PP020 [T]	86.00	47.76	2735266	5727087
Pseudomonas sichuanensis WCHPs060039 [T]	85.98	47.14	2699896	5727087
Pseudomonas fakonensis COW40 [T]	85.98	46.90	2685844	5727087
Pseudomonas xantholysinigenes RW9S1A [T]	85.97	45.96	2632070	5727087
Pseudomonas oryziphila 1257 [T]	85.94	46.93	2687613	5727087
Pseudomonas xanthosomae COR54 [T]	85.93	47.14	2699621	5727087
Pseudomonas mosselii DSM 17497 [T]	85.93	47.20	2703421	5727087
Pseudomonas maumuensis COW77 [T]	85.88	45.93	2630725	5727087
Pseudomonas asiatica RYU5 [T]	85.86	43.95	2517026	5727087
Pseudomonas entomophila L48 [T]	85.85	46.57	2667022	5727087
Pseudomonas shirazica VM14 [T]	85.85	44.06	2523229	5727087
Pseudomonas plecoglossicida NBRC 103162 [T]	85.83	41.76	2391786	5727087
Pseudomonas anuradhapurensis RD8MR3 [T]	85.83	42.90	2457160	5727087
Pseudomonas inefficax JV551A3 [T]	85.83	43.99	2519466	5727087
Pseudomonas soli LMG 27941 [T]	85.70	45.25	2591353	5727087
Pseudomonas capeferrum WCS358 [T]	85.68	43.58	2495918	5727087
Pseudomonas vlassakiae RW4S2 [T]	85.63	42.44	2430699	5727087
Pseudomonas urethralis BML-PP042 [T]	85.62	43.15	2471461	5727087

Note: The "T" in square brackets represents the type strain.

Table S3. Several putative PQQ-dependent dehydrogenases in *Pseudomonas* sp. SD17-1

Name of subject enzyme	E value	Identities with mature DepA (%)	Coverage (%)	Accession number	Proenzyme (aas)	Signal peptide	Family
ADH1	3E-51	28	26.3	WEJ19651.1	697	1–18	PQQ_like domain of the quinohemoprotein alcohol dehydrogenase (type II), cd10279 PQQ-ADH II
ADH2	7E-44	27	25.6	WEJ19787.1	690	1–22	PQQ_like domain of the quinohemoprotein alcohol dehydrogenase (type II), cd10279 PQQ-ADH II
ADH3	1E-09	26	15.0	WEJ19927.1	808	No	Membrane-bound PQQ-dependent glucose dehydrogenase, cd10280 PQQ_mGDH
ADH4	8E-51	29	26.9	WEJ19989.1	718	1–29	PQQ_like domain of the quinohemoprotein alcohol dehydrogenase (type II), cd10279 PQQ-ADH II
ADH5	4E-08	50	5.7	WEJ20631.1	806	No	Membrane-bound PQQ-dependent glucose dehydrogenase, cd10280 PQQ_mGDH
ADH6	7E-15	23	24.5	WEJ21343.1	786	No	Membrane-bound PQQ-dependent glucose dehydrogenase， cd10280 PQQ_mGDH
ADH7	1E-07	40	6.4	WEJ24222.1	811	No	Membrane-bound PQQ-dependent glucose dehydrogenase，cd10280 PQQ_mGDH

Table S4. Primer sequence and PCR reaction program

Candidate genes	Forward primer (5′-3′)	Reverse primer (5′-3′)	Thermocycling conditions
17DH1	*AGGAGATATACCATG*CAGTCGGACCTTCTGGAGAACA	*GTGGTGGTGGTGGTG*CTTCTGCGGAAGGGCGAAG	98°C/2 min; 30×(98°C/10s, 63°C/30s, 72°C/1min); 72°C/4min
17DH2	*AGGAGATATACCATG*CAGTCGGATCTGCTGGAGAACA	*GTGGTGGTGGTGGTG*CTTCTGCGGCAGGGCG	98°C/2 min; 30×(98°C/10s, 63°C/30s, 72°C/1min); 72°C/4min
17DH3	*AGGAGATATACCATG*CAGGGCGTCCGCGAAA	*GTGGTGGTGGTGGTG*CTGCTTGGGCAGCTTGAAGAC	98°C/2 min; 30×(98°C/10s, 63°C/30s, 72°C/1min); 72°C/4min
17DH4	*AGGAGATATACCATG*CAGGAAAACCCCATCGACAGC	*GTGGTGGTGGTGGTG*CTTAGCGTCGGGCAGTGC	98°C/2 min; 30×(98°C/10s, 63°C/30s, 72°C/1min); 72°C/4min
Vector	CATGGTATATCTCCTTCTTAAAGTTAAACAAAATTATTTCTAGAGG	CACCACCACCACCACCACT	98°C/4 min; 30×(98°C/30s, 68°C/30s, 72°C/2min); 72°C/10min

Note: The italicized bases in the primer sequence indicate homology to the expression vector sequence.

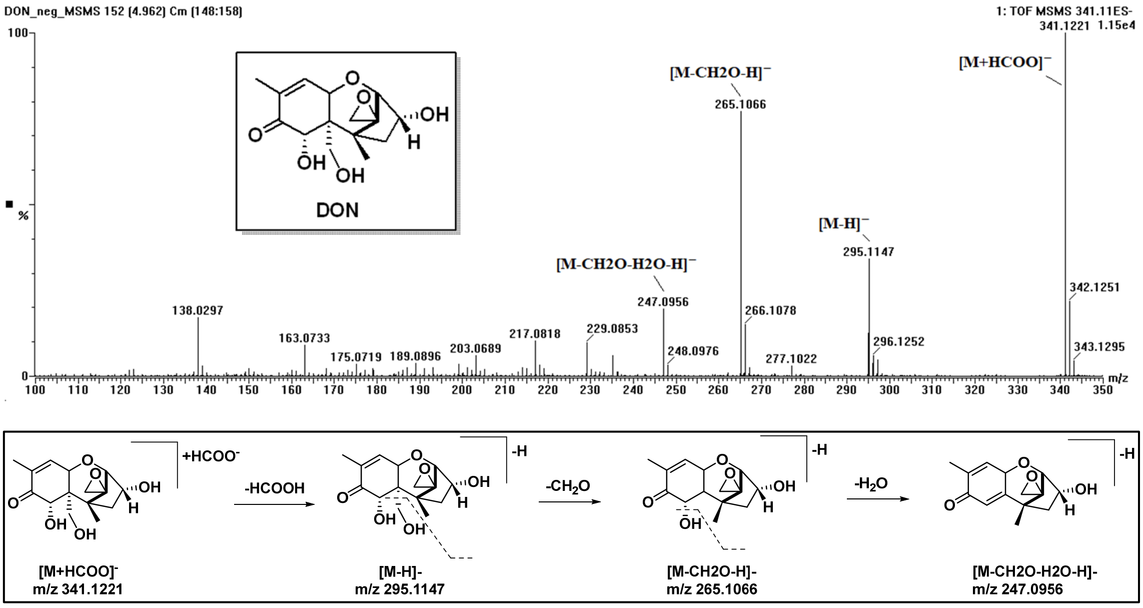

Figure S1. The proposed product ions in the fragmentation of m/z 341.1221 ([M+HCOO]-) for DON.

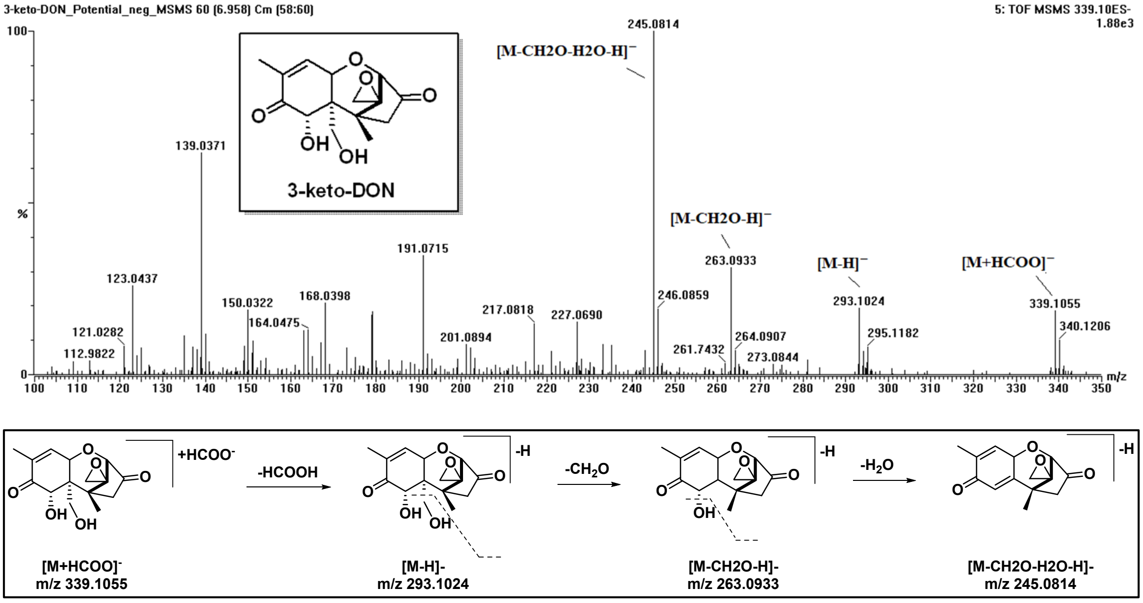

Figure S2. The proposed product ions in the fragmentation of m/z 339.1055 ([M+HCOO]-) for metabolite.
